# Volumetric and Linear Imaging Response After Stereotactic Radiotherapy for Vestibular Schwannoma

**DOI:** 10.7759/cureus.98252

**Published:** 2025-12-01

**Authors:** Eduardo M Rocha, Pedro T Costa, Mavilde Arantes, Artur Aguiar, Andreia Pires, Sofia Conde

**Affiliations:** 1 Radiation Oncology, Instituto Português de Oncologia do Porto Francisco Gentil, Porto, PRT; 2 Neuroradiology, Instituto Português de Oncologia do Porto Francisco Gentil, Porto, PRT

**Keywords:** radiographic response, stereotactic fractionated radiotherapy, stereotactic hypofractionated radiotherapy, stereotactic radiosurgery, stereotactic radiotherapy, treatment toxicity, tumor control, vestibular schwannoma

## Abstract

Introduction: Vestibular schwannomas (VS) are benign tumors of the vestibulocochlear nerve. Advances in magnetic resonance imaging (MRI) have increased the detection of smaller lesions, shifting management toward functional preservation. Stereotactic radiotherapy (SRT) delivered as single-fraction stereotactic radiosurgery (SRS), hypofractionated stereotactic radiotherapy (HFSRT), or conventionally fractionated stereotactic radiotherapy (FSRT) achieves high tumor control, but standardized progression criteria and regimen-stratified outcomes remain underexplored.

Methods: We retrospectively reviewed adults with VS treated with SRT (2013-2022). Baseline features, symptoms, and treatment variables were collected. Tumor control was assessed volumetrically (≥20% increase) and linearly (maximum linear diameter (MLD) ≥2 mm) at 12 months, 24 months, and five years. Statistical analyses explored associations with clinical and imaging factors.

Results: Among 143 patients (median age 58; 84 (59%) female), Koos stage III-IV predominated, present in 104 (73%) patients, 44 (31%) had prior surgery, 86 (60%) had cystic component, and 17 (12%) had T2-weighted hypersignal. Regimens were SRS in 23 (16%), HFSRT in 59 (41%), and FSRT in 61 (43%) patients. The median follow-up was 6.5 years. Baseline symptoms were frequent, reported in 141 (99%) patients, mainly hearing loss (127, 89%) and tinnitus (75, 52%). Volumetric progression declined over time, documented in 25 (22%) patients at 12 months, 18 (15%) at 24 months, and 14 (17%) at five years (p=0.030), while linear rates were stable, occurring in 16 (14%) patients at 12 months, 15 (13%) at 24 months, and 10 (12%) at five years. Both measures correlated strongly (ρ=0.76-0.87). Volumetric progression predicted higher clinical worsening, especially hearing decline at 12 months and five years (p<0.05). Outcomes were comparable at five years across regimens, though SRS showed earlier pseudoprogression at 24 months. Salvage surgery or re-irradiation occurred in eight patients (6%).

Conclusion: SRT achieved durable control of VS across SRS, HFSRT, and FSRT in this single-institution cohort, with few salvage interventions. The three-axis volumetric criterion (20% increase) outperformed the MLD 2 mm threshold and correlated strongly with contoured gross tumor volume (GTV). Early progression subsided by 24 months, and stability at 12 and 24 months predicted five-year control. Between-regimen divergence at 24 months was consistent with SRS-driven pseudoprogression, with five-year progression remaining similar across regimens. Baseline imaging offered limited prognostic value, whereas clinical worsening, mainly hearing decline, correlated with volumetric progression. Annual MRI after year 1 is generally adequate, with tighter intervals reserved for selected patients. Prospective, audiometry-standardized studies are warranted for validating hearing-related endpoints.

## Introduction

Vestibular schwannomas (VS), also known as acoustic neuromas, are benign tumors of the vestibular portion of the VIII cranial nerve, accounting for approximately 8% of intracranial tumors [1]. The increased use of high-resolution magnetic resonance imaging (MRI) has led to a higher detection of smaller tumors [2]. This epidemiologic shift resulted in management strategies that prioritize functional preservation and the minimization of treatment-related morbidity. Current options include watch-and-wait, surgical resection, and stereotactic radiotherapy (SRT) [3,4]. Radiotherapy (RT) is widely used for up to medium-sized tumors, achieving long-term control rates above 90% with low rates of facial or trigeminal nerve complications [3,5]. The most frequently used stereotactic regimens include single-fraction stereotactic radiosurgery (SRS), hypofractionated stereotactic radiotherapy (HFSRT), and conventionally fractionated stereotactic radiotherapy (FSRT). Comparative series and systematic reviews have not demonstrated a definitive superiority of one regimen over another, with selection often reflecting tumor size, proximity to critical structures, and institutional preference [6-8].

Imaging control remains the oncologic foundation of management, though hearing preservation is often emphasized [1,7]. Progression definitions vary. A ≥2 mm increase in maximum linear diameter (MLD) is simple and familiar, but one-dimensional is susceptible to misclassifying post-RT pseudoprogression, whereas a volumetric criterion, such as a ≥20% increase, better reflects true tumor progression and may reduce variability in reported outcomes [1,7,9,10]. These differing definitions likely contribute to variability in reported progression across studies.

Baseline characteristics (tumor size, Koos stage, T1-weighted imaging (T1W), cystic component, T2-weighted (T2W) signal, and prior surgery) may influence clinical presentation and radiologic tumor response [11,12]. However, few studies provide regimen-stratified outcomes with long follow-up while applying standardized response criteria and incorporating patient-reported symptoms.

This single-institution study evaluated SRT for VS (SRS, HFSRT, FSRT) with extended follow-up. The primary endpoint was local control using a volumetric increase at 24 months and five years. Secondary objectives were to compare this with an MLD increase, quantify correlation and agreement between volumetric and linear measures across 12 months, 24 months, and five years, and explore associations with regimen, baseline features, and patient-reported symptoms.

This work was presented as an oral presentation at the CNO 2025 Beyond Limits, 22º Congresso Nacional de Oncologia.

## Materials and methods

A retrospective cohort study was conducted at the Instituto Português de Oncologia do Porto Francisco Gentil, a specialized tertiary center in Porto, Portugal. Inclusion criteria were age ≥18 years, radiologically confirmed VS, completion of SRT at our institution between January 2013 and December 2022, availability of planning MRI, and at least one post-treatment MRI suitable for analysis. The only exclusion criterion was the absence of follow-up imaging; no patients were excluded based on baseline clinical or imaging characteristics. Clinical follow-up data were collected up to July 2025. Data were obtained from institutional databases and electronic medical records. Ethical approval was obtained from the Ethics Committee for Health of the Instituto Português de Oncologia do Porto Francisco Gentil (approval number: CES. 93/023).

Baseline data included age, sex, laterality, NF2 status, and history of prior surgery or RT. Pre-treatment symptom status (hearing loss, tinnitus, ataxia, facial weakness, trigeminal dysfunction, vertigo, and headache) was documented. Standardized audiometric assessments were inconsistently available, constituting an inherent methodological limitation for interpreting hearing outcomes in this retrospective cohort. Symptoms (new or worsened) were obtained from patient reports, corroborated by physician documentation. Post-RT interventions, including surgery and re-irradiation, were also collected. Treatment variables included RT regimen, prescribed dose, fractionation, and treatment duration. Biological effective dose (BED) was calculated assuming α/β=2 [13].

All patients underwent a planning 3D MRI with 1 mm slices. Tumor delineation was performed using the Varian Eclipse (Palo Alto, California, United States) or Brainlab software (Munich, Germany). Contrast-enhanced T1W sequences were used for gross tumor volume (GTV) contouring, linear measuring, Koos stage, and assessment of cystic component. For linear assessment, MLD was defined as the largest measurable axial tumor dimension. At the same axial slice, the orthogonal projection was measured. The cranio-caudal extent was measured on coronal (non-oblique) reconstruction. The intracanalicular portion was included in the MLD if it was visible on the same slice as the maximal diameter and formed part of that measurement [14]. Linear progression was defined as MLD ≥2 mm compared to baseline. For volumetric assessment, a three-axis product (ABC/3) was calculated using the axial MLD (A), its maximal orthogonal diameter measured on the same axial slice (B), and the corresponding cranio-caudal diameter measured on the coronal plane (C) [15].** **This method was cross-validated against the respective GTV in preliminary analyses and showed very high agreement, supporting its use in this cohort. For consistency across follow-up, tumor response was expressed as the volume ratio (follow-up/baseline), with progression defined by ≥20% increase, minimizing potential formula-estimation errors compared with surrogate volume methods. Follow-up imaging was evaluated at 12 months, 24 months, and five years post-treatment. A margin of within three months was allowed for each timepoint, with MRI slice thickness ≤3 mm. Imaging outside these windows was excluded from the respective timepoint analysis. The same tumor metrics (MLD, orthogonal, and coronal projections) were measured at each timepoint by a single observer to ensure consistency. Analyses used available cases at each timepoint without imputation.

Normality (Shapiro-Wilk) and homoscedasticity (Levene) were assessed. Because distributions were non-normal, continuous data are reported as median, interquartile range (IQR), and range and compared with Mann-Whitney U or Kruskal-Wallis with Dunn-Holm adjustment. Associations used Spearman's rank correlation. Categorical data used χ² or Fisher's exact (with Monte Carlo for 3×2 tables). Within-patient longitudinal change in continuous volumetric and linear measurements was tested with Friedman and Wilcoxon signed-rank post hoc. Progression rates used Cochran's Q with McNemar post hoc, while intra-patient calculations used positive and negative predictive values (PPV and NPV) and Cohen's κ. Regimens (SRS, HFSRT, FSRT) were analyzed in parallel and stratified by baseline features (Koos stage, cystic component, T2W signal, prior surgery, and symptoms). Analyses were performed in JASP (0.95.0.0) (JASP Team, University of Amsterdam, Amsterdam, The Netherlands) and Python (3.11) (Python Software Foundation, Fredericksburg, Virginia, United States). Two-sided p<0.05 denoted significance.

## Results

A total of 143 adult patients with VS treated with SRT were included. No baseline demographic, imaging, or treatment data were missing. The median age at the start of treatment was 58 years (IQR 49-68; range 18-87), and 58.7% of patients were female. The tumor side was evenly distributed (52.4% left). NF2 mutation was present in six patients (4.2%). Prior surgery had been performed in 30.8%, while no patients had received prior RT. The most frequent Koos stage was IV (37.1%), followed by III (35.7%), II (24.5%), and I (2.8%). On baseline MRI, cystic component was present in 60.1% of tumors and T2W hypersignal in 11.9%. Median GTV was 1.8 cc (IQR 0.9-3.65; range 0.1-13.0), with corresponding MLD 21 mm (IQR 17.1-25.5; range 8.9-49), orthogonal axis 14.1 mm (IQR 11.3-18.9; range 3.8-30), and coronal axis 15.1 mm (IQR 11.0-20.9; range 5-33). The three-axis formula (ABC/3) had a median volume of 1.47 cc (IQR 0.76-3.21; range 0.1-12.0). Baseline demographic, imaging, and treatment characteristics are summarized in Table 1.

**Table 1:** Baseline demographic, imaging, and treatment characteristics No missing data for demographic, imaging, or treatment variables. Orthogonal: axial diameter perpendicular to the maximum linear diameter. Coronal: cranio-caudal diameter measured on the coronal plane. (A×B×C)/3: three-axis surrogate volume using axial MLD (A), orthogonal diameter (B), and coronal diameter (C). IQR: interquartile range; GTV: gross tumor volume; MLD: maximum linear diameter; RT: radiotherapy; SRS: stereotactic radiosurgery; HFSRT: hypofractionated stereotactic radiotherapy; FSRT: fractionated stereotactic radiotherapy

Patient characteristics		N=143
Demographics	Age (years), median (IQR)	57.9 (48.6-68.3)
	Female sex, n (%)	84 (58.7%)
	Tumor laterality left, n (%)	75 (52.4%)
	NF2 mutation, n (%)	6 (4.2%)
	Prior surgery, n (%)	44 (30.8%)
	Prior RT, n (%)	0 (0%)
Imaging	Koos stage, n (%)	I: 4 (2.8%)
		II: 35 (24.5%)
		III: 51 (35.7%)
		IV: 53 (37.1%)
	T1W cystic component present, n (%)	86 (60.1%)
	T2W hypersignal present, n (%)	17 (11.9%)
	GTV (cc), median (IQR)	1.8 (0.9-3.65)
	MLD (mm), median (IQR)	21 (17.1-25.5)
	Orthogonal (mm), median (IQR)	14.1 (11.3-18.85)
	Coronal (mm), median (IQR)	15.1 (10.95-20.85)
	Baseline ((A×B×C)/3) (cc), median (IQR)	1.47 (0.76-3.21)
RT regimen	SRS, n (%)	23 (16.1%)
	HFSRT, n (%)	59 (41.3%)
	FSRT, n (%)	61 (42.7%)
Follow-up	Follow-up time (years), median (IQR)	6.5 (3.8-8.6)

The chosen RT regimen was SRS in 16.1% (median dose 14 Gy; range 12-18 Gy; single fraction), HFSRT in 41.3% (median 30 Gy in five fractions; range 25-42 Gy; 5-14 fractions), and FSRT in 42.7% (median 50 Gy; range 45-54 Gy; 25-28 fractions). Overall median BED(α/β=2) was 108 Gy. Detailed prescription parameters, including BED and EQD2, are shown in Table 2.

**Table 2:** Radiotherapy prescription characteristics Values are presented as median (IQR). Percentages refer to the proportion of the full cohort (N=143). EQD2: equivalent dose in 2 Gy fractions; BED: biologically effective dose; SRS: stereotactic radiosurgery; HFSRT: hypofractionated stereotactic radiotherapy; FSRT: fractionated stereotactic radiotherapy

Regimen	n (%)	Dose (Gy)	Fractions	Dose per fraction (Gy)	EQD2 (Gy)	BED (Gy)
Overall	143 (100%)	30 (14-50)	5 (1-25)	2.0 (2.0-14)	54 (50-60)	108 (100-120)
SRS	23 (16%)	14 (12.8-14)	1 (fixed)	14	56 (42-90)	112 (84-180)
HFSRT	59 (41%)	30 (27.5-30)	5 (5-5)	6.0 (5.0-6.0)	60 (43.8-60)	120 (87.5-120)
FSRT	61 (43%)	50 (50-50.4)	25 (25-28)	2.0 (1.8-2.0)	50 (42.8-54)	100 (85.5-108)

The median follow-up was 6.5 years (IQR: 3.8-8.6; range: 0-11.6). Cystic tumors had significantly larger volumes than solid tumors (median GTV 2.7 vs. 1.0 cc; p<0.001). Similarly, patients with prior surgery had larger tumors (3.05 vs. 1.5 cc; p<0.001). No significant GTV differences were observed by T2W signal (p=0.294) or RT regimen (p=0.085), although volumes tended to be larger with higher fractionation (FSRT 2.5 cc, HFSRT 2.0 cc, SRS 1.5 cc). Detailed comparisons are presented in Table 3.

**Table 3:** Tumor volume associations Values are median (IQR). The Kruskal-Wallis test (H) was used for comparison across RT regimens. The Mann-Whitney U test (U) was used for two-group comparisons (T1W solid vs. cystic component, T2W signal, and prior surgery). RT: radiotherapy; SRS: stereotactic radiosurgery; HFSRT: hypofractionated stereotactic radiotherapy; FSRT: fractionated stereotactic radiotherapy; T1W: T1-weighted; T2W: T2-weighted; (+): hypersignal; (-): hypersignal absent

Comparison	Category	GTV (cc): median, (IQR)	Test statistic value	P-value
RT regimen	SRS	1.5 (0.8-1.7)	H=4.932	0.085
	HFSRT	2.0 (1.05-3.35)		
	FSRT	2.5 (0.8-5.5)		
T1W	Solid	1.0 (0.7-2.3)	U=3570	<0.001
	Cystic	2.7 (1.5-4.8)		
T2W	(+)	2.3 (1.5-3.7)	U=902.5	0.294
	(-)	1.7 (0.83-3.6)		
Prior surgery	Yes	3.05 (1.5-6.6)	U=1406	<0.001
	No	1.5 (0.7-2.9)		

The three-axis surrogate volume (ABC/3) showed a strong correlation with the contoured GTV (ρ=0.982; p<0.001), presented in Table 4.

**Table 4:** Correlation between GTV and three-axis formula Spearman's rank-order correlation test. (A×B×C)/3: three-axis surrogate volume using axial MLD (A), orthogonal diameter (B), and coronal diameter (C). MLD: maximum linear diameter; GTV: gross tumor volume

Variable	Spearman's ρ	P-value
GTV vs. (A×B×C)/3 (cc)	0.982	<0.001

At baseline, nearly all patients (98.6%) reported at least one symptom (median burden 3). Hearing loss (88.8%) and tinnitus (52.4%) were the most common, followed by ataxia (39.9%), facial weakness (29.4%), vertigo (25.9%), trigeminal dysfunction (18.2%), and headache (14.7%). Individual symptom prevalence did not statistically differ by cystic component or T2W signal (p>0.05). In contrast, prior surgery was associated with higher rates of facial weakness (72.7% vs. 10.1%; p<0.001), trigeminal symptoms (34.1% vs. 11.1%; p=0.002), and hearing loss (97.7% vs. 84.8%; p=0.023) and with lower prevalence of tinnitus (31.8% vs. 61.6%; p=0.001) and vertigo (9.1% vs. 33.3%; p=0.002). Symptom burden did not significantly differ across RT regimens (p=0.738), cystic component (p=0.440), or T2W signal (p=0.872). Tumor volume was not significantly correlated with symptom burden (p=0.116). Symptom burden was also higher in previously operated patients (p=0.008). Symptom prevalence did not significantly differ across RT regimens, except for vertigo, which was more frequent in HFSRT than in SRS (32.2% vs. 8.7%; p=0.046).

**Figure 1:**
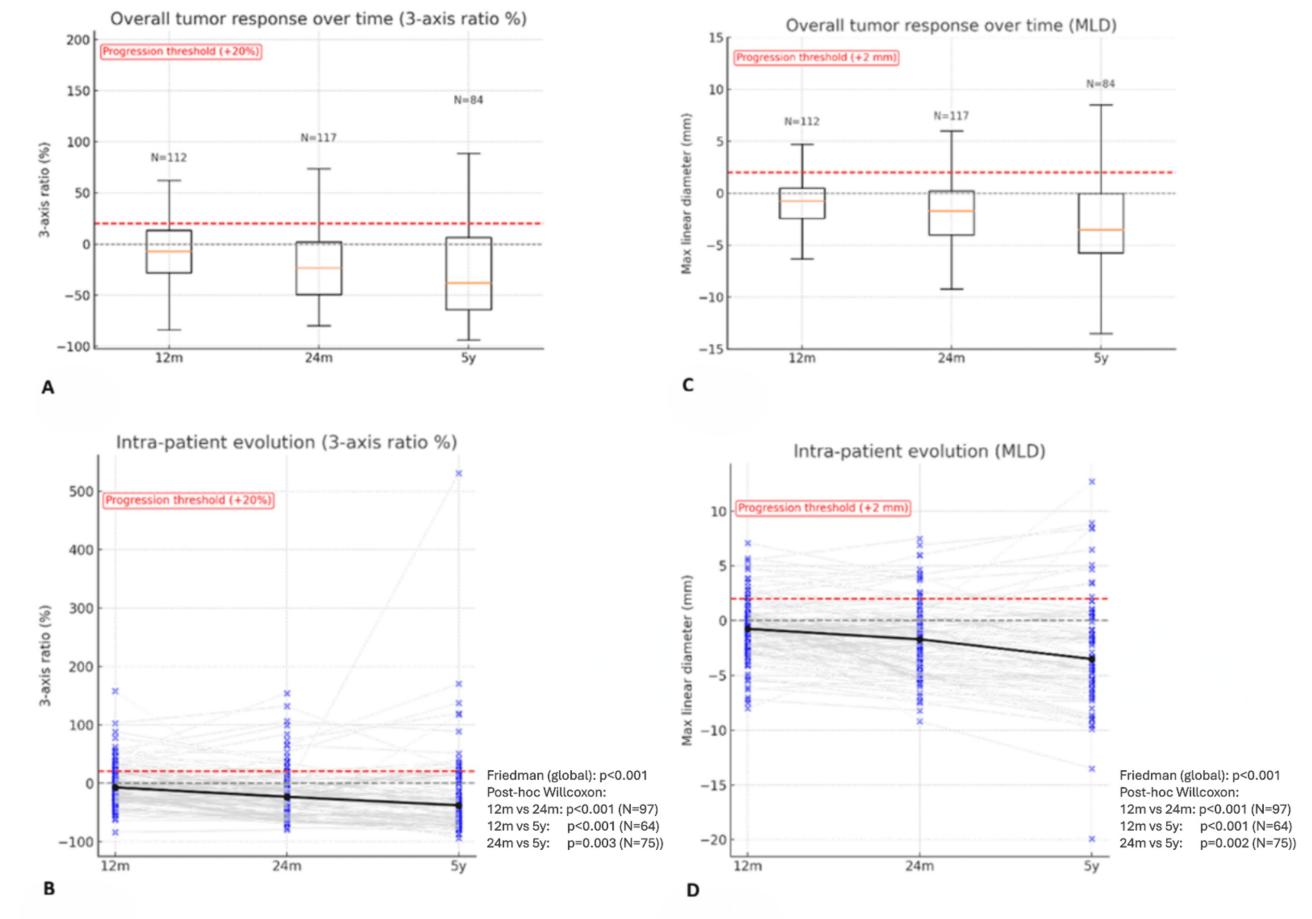
Continuous local control and intra-patient analysis using volumetric (three-axis ratio %) and linear (MLD mm) measures at 12 months, 24 months, and five years Overall volumetric response (three-axis ratio; panels A-B) and linear response (MLD; panels C-D) are shown at 12 months, 24 months, and five years. Panels A and C display the overall distributions at each timepoint (unpaired data) and are shown descriptively. Red dashed lines indicate the progression thresholds (+20% for volumetric ratio and +2 mm for MLD). Grey dashed lines indicate the 0% (three-axis ratio) or 0 mm (MLD) reference level. Panels B and D display intra-patient repeated-measures data across timepoints (paired data); the black solid line represents the cohort median trajectory over time. Formal statistical testing for intra-patient repeated-measures data (panels B and D) was performed using the Friedman test with post hoc Wilcoxon signed-rank tests, and the corresponding p-values and paired sample sizes (N) are shown within these panels. MLD: maximum linear diameter; m: months; y: years

Follow-up imaging was available for 112 patients at 12 months, 117 at 24 months, and 84 at five years. Using the three-axis volumetric criterion, overall progression rates were 22.3% at 12 months, 15.4% at 24 months, and 16.7% at five years, with a significant decrease across time (p=0.030). Pairwise analysis confirmed fewer events between 12 and 24 months (p=0.021), while differences between 12 months and five years and 24 months and five years were not significant. In contrast, MLD ≥2 mm progression rates remained stable over time (14.3%, 12.8%, and 11.9% at 12 months, 24 months, and five years; p=0.905; all pairs p=1.000). Continuous analyses confirmed overall decline with time (p<0.001). The median volumetric response decreased by -7.2% at 12 months, -23.3% at 24 months, and -37.8% at five years, while MLD declined by -0.8 mm, -1.7 mm, and -3.5 mm (all pairwise comparisons p<0.01). Both distributions are illustrated in Figure 1A (three-axis) and Figure 1C (MLD). Continuous intra-patient analysis corroborated a significant reduction in both volumetric ratio and MLD across follow-up (both p<0.001; Figure 1B and Figure 1D). Pairwise Wilcoxon tests showed progressive shrinkage between 12 and 24 months (p<0.001; N=97), 12 months and five years (p<0.001; N=64), and 24 months and five years (p=0.003; N=75).

Intra-patient progression agreement was modest. Between 12 and 24 months, almost all non-progressions remained stable (NPV=96%), while 41% of progressions persisted (PPV=41%), yielding moderate agreement (κ=0.44). From 12 months to five years, agreement was weaker (κ=0.32), characterized by high NPV (94%) but low PPV (33%). In contrast, from 24 months to five years, both PPV (78%) and NPV (89%) were higher, corresponding to moderate-good agreement (κ=0.54). Volumetric tumor control across clinical timepoints and subgroups are summarized in Table 5.

**Table 5:** Progression rates using volumetric measures (three-axis ratio %) at 12 months, 24 months, and five years Proportions are presented as n/N (%). Progression defined as ≥20% volumetric increase. Global p-values and test statistics for three-group comparisons were obtained using the chi-squared test (χ²). P-values for two-group comparisons were calculated using two-sided Fisher's exact test. RT: radiotherapy; SRS: stereotactic radiosurgery; HFSRT: hypofractionated stereotactic radiotherapy; FSRT: fractionated stereotactic radiotherapy; T1W: T1-weighted; T2W: T2-weighted; (+): hypersignal; (-): hypersignal absent

Progression	Category	12 months	χ²	P-value	24 months	χ²	P-value	5 years	χ²	P-value
Overall	N=143	25/112 (22.3%)	-	-	18/117 (15.4%)	-	-	14/84 (16.7%)	-	-
RT regimen	SRS	5/17 (29.4%)	1.04	0.593	5/17 (29.4%)	6.24	0.044	3/14 (21.4%)	0.29	0.866
	HFSRT	10/41 (24.4%)			10/52 (19.2%)			6/37 (16.2%)		
	FSRT	10/54 (18.5%)			3/48 (6.2%)			5/33 (15.2%)		
Koos stage	I-II	9/28 (32.1%)	-	0.206	9/34 (26.5%)	-	0.025	4/22 (18.2%)	-	0.877
	III-IV	16/84 (19%)			9/83 (10.8%)			10/62 (16.1%)		
T1W	Cystic	13/67 (19.4%)	-	0.488	13/70 (18.6%)	-	0.302	10/44 (22.7%)	-	0.149
	Solid	12/45 (26.7%)			5/47 (10.6%)			4/40 (10%)		
T2W	(+)	2/12 (16.7%)	-	1	1/14 (7.1%)	-	0.692	2/12 (16.7%)	-	1
	(-)	23/100 (23%)			17/103 (16.5%)			12/72 (16.7%)		
Prior surgery	No	18/80 (22.5%)	-	1	9/81 (11.1%)	-	0.093	8/64 (12.5%)	-	0.088
	Yes	7/32 (21.9%)			9/36 (25%)			6/20 (30%)		

**Figure 2:**
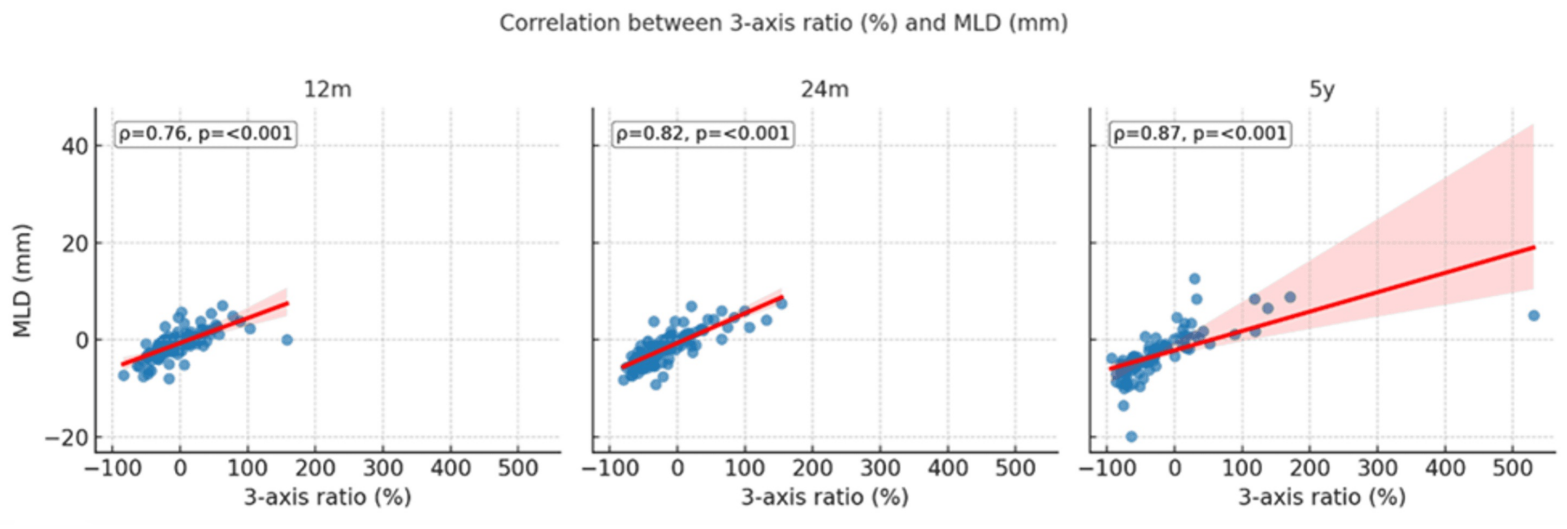
Correlation between volumetric (three-axis ratio %) and linear (MLD mm) measures at 12 months, 24 months, and five years Scatter plots show Spearman's correlations (ρ) with regression lines and 95% confidence intervals. All correlations were significant (p<0.001). MLD: maximum linear diameter; m: months; y: years

Volumetric and linear response measures were strongly correlated at all timepoints (ρ=0.76-0.87; p<0.001; Figure 2). Agreement between three-axis volumetric and MLD-based progression criteria remained high (85-92%), with early discordances at 12 months driven primarily by three-axis progression (p=0.049), while no significant differences were seen at 24 months or five years. Baseline GTV showed no correlation with three-axis volumetric response (all p>0.05), although an inversely proportional weak correlation was found with MLD reduction at 12 months (ρ=-0.22; p=0.022) and five years (ρ=-0.25; p=0.020).

Regimen-stratified outcomes showed comparable volumetric progression rates at 12 months (SRS 29.4%, HFSRT 24.4%, FSRT 18.5%; p=0.593) and at five years (21.4%, 16.2%, 15.2%; p=0.866), but differed significantly at 24 months (29.4%, 19.2%, 6.2%; p=0.044). Continuous analysis confirmed the progressive decline across regimens, with median changes at 12 months, 24 months, and five years of SRS (+7.8% » -19.9% » -46.7%), HFSRT (-11.7% » -22.1% » -38%), and FSRT (-9.9% » -24% » -37.7%), without significant differences between groups (all p>0.5). By the Koos stage, early tumors (I-II) showed a nonsignificant higher progression trend than stage III-IV at 12 months (32.1% vs. 19%; p=0.206) and significantly higher rates at 24 months (26.5% vs. 10.8%; p=0.025), but outcomes converged by five years (18.2% vs. 16.1%; p=0.877). Cystic component was not associated with significant progression rates at any timepoint, despite the nonsignificant trend toward higher rates (five years: 22.7% vs. 10%; p=0.149). Continuous volumetric changes were consistent with the findings. T2W hypersignal was likewise not associated with local control, with progression rates comparable across all timepoints (16.7% at five years for both groups; all p≥0.69). Continuous data suggests a higher response at 24 months and five years, although not significant (five years: -56.4% vs. -35.5%; p=0.147). Prior surgery was not associated with differences in progression at 12 months (22.5% vs. 21.9%; p=1.000), although a nonsignificant trend toward higher progression was observed at 24 months (25% vs. 11.1%; p=0.093) and five (30% vs. 12.5%; p=0.088). Continuous volumetric findings supported this pattern, showing significantly smaller reductions in patients with prior surgery (five years: -14% vs. -45.5%; p=0.037).

Clinical worsening was significantly associated with volumetric radiographic progression. At 12 months, overall worsening was more frequent in the progression group (48% vs. 25.3%; p=0.047), driven by higher rates of hearing decline (24% vs. 6.9%; p=0.025). By five years, both overall worsening (64.3% vs. 25.7%; p=0.010) and hearing loss (35.7% vs. 10%; p=0.025) remained significantly more frequent in progressors, whereas other symptoms showed no association with progression (p>0.05). Across RT regimens, post-RT symptom worsening did not differ significantly (p>0.05), though a nonsignificant trend toward more facial weakness was observed in the HFSRT group (0% SRS, 15.3% HFSRT, 4.9% FSRT; p=0.07), with post hoc pairwise comparisons confirming no significant differences (all p>0.05). New-onset post-RT symptoms were uncommon. Hearing loss was the most frequent (50% among patients with baseline hearing preserved), followed by ataxia (10.5%), facial weakness (7.9%), trigeminal dysfunction (6.8%), headache (3.3%), tinnitus (1.5%), and vertigo (0.9%). No significant differences across RT regimens (all p>0.1). Post-RT symptom worsening was not associated with cystic component, T2W signal, or prior surgery (all p>0.2). Patients who experienced post-RT symptom worsening had a significantly larger baseline GTV compared with those who remained stable (2.5 vs. 1.7 cc; p=0.038).

Post-RT interventions (seven surgeries, one re-irradiation) occurred in eight patients (5.6%), without significance across regimens (p=0.636). The median time to intervention was 3.8 years (range 1.4-8.7). Loss to follow-up was 21.7% at 12 months (n=31), 14% at 24 months (n=20), and 10.5% at five years (n=15). Most cases were due to MRI performed outside the protocol window (23, 14, and seven patients at each timepoint). Smaller numbers reflected unrelated death, limited imaging access, surgery before follow-up, or true loss to follow-up.

## Discussion

This retrospective cohort of 143 patients, with a median follow-up of 6.5 years (up to 11.6 years), represents one of the most comprehensive single-center series with prolonged follow-up of VS treated with SRT (SRS, HFSRT, FSRT). There were no missing baseline demographic, imaging, and treatment data. The cohort had a median age of 58 years, slight female predominance, laterality evenly distributed, and low prevalence of NF2-related tumors, aligning with the expected distribution of sporadic disease [1,16,17]. Prior surgery was performed in nearly one-third of patients, consistent with other institutional series, while the absence of previous RT reflects the rarity of re-irradiation in this setting [18]. In our study, 73% of tumors were classified as Koos stage III-IV, reflecting the increasing use of SRT for larger tumors, in line with recent multi-institutional studies [19]. Cystic component was present in 60% of tumors, notably higher than the 20-40% typically reported in SRT cohorts, whereas T2W hypersignal was uncommon (12%), but less consistently described in the literature [11,20]. The median tumor size (GTV and MLD) was consistent with contemporary SRT series, where median volumes typically range 1.2-2.6 cm³ and MLDs are <30 mm. The strong correlation between GTV and the three-axis formula (ABC/3), also reported in prior studies, supports its validity for baseline volumetric assessment [15]. The distribution of RT regimens (SRS, HFSRT, and FSRT) mirrors current practice [5].

Cystic lesions had significantly larger volumes, consistent with multi-institutional studies. Similarly, tumors in patients with prior surgery were also larger (p<0.001), which aligns with reports indicating that recurrent or residual tumors tend to be larger and more complex. No significant differences in tumor volume were observed by T2W signal or RT regimen, although there was a trend toward larger volumes in patients treated with higher fractionation regimens. This pattern is expected, as FSRT is often selected for bulkier tumors or those in proximity to critical structures, but the lack of statistical significance suggests that other factors may also influence regimen selection [1,5,21,22].

Nearly all patients reported at least one symptom at baseline (median 3), with hearing loss (88.8%) and tinnitus (52.4%) being the most common, also consistent with other large series. Other symptoms were also frequently reported, reflecting the spectrum of cranial nerve and cerebellar involvement described in the literature. No association between cystic component or T2W signal was found, confirming that symptom burden is not reliably predicted by tumor imaging characteristics. In contrast, prior surgery was associated with higher rates of facial and trigeminal symptoms, consistent with reports that surgical intervention increases the risk of cranial nerve morbidity [1,23,24].

Longitudinal assessment showed that volumetric progression rates declined with time, while linear criteria based on maximum diameter remained largely stable, reinforcing evidence that volumetric analysis is more sensitive for capturing post-RT tumor dynamics. Continuous measures confirmed progressive shrinkage, consistent with prior reports that most tumors remain stable or regress after SRT. Once stability was observed at 12 months, subsequent progression was uncommon, with NPVs exceeding 90%. By contrast, early apparent enlargements were often transient, underscoring the risk of misclassifying pseudoprogression. Stability beyond 24 months was a reliable predictor of durable control, suggesting that MRI intervals shorter than one year add cost and burden without clear clinical benefit, whereas annual surveillance after the first year appears an appropriate and efficient strategy. These findings support volumetric analysis as the preferred metric for follow-up and emphasize the importance of long-term monitoring to detect the minority of late progressors [25-27].

Volumetric and linear measures showed strong, time-strengthening concordance, in line with evidence. The high agreement between three-axis volumetric and MLD criterion confirms prior reports: small linear increases often correspond to substantial volumetric change, while early discordances at 12 months driven by volumetric progression reflect pseudoprogression and highlight the greater sensitivity of volumetric analysis, especially for smaller lesions. The absence of associations with baseline tumor size or symptoms is consistent with studies showing that initial volume alone does not predict post-SRT behaviour [19,28].

Regimen-stratified outcomes were comparable at 12 months and five years, although with a significant difference at 24 months (p=0.044) driven by higher apparent progression after SRS. This transient progression is consistent with reports of post-SRS. Continuous volumetrics declined across all regimens without between-group differences. By the Koos stage, early tumors (I-II) also showed higher progression only at 24 months, with continuous volumetric changes comparable by stage. Cystic component and T2W hypersignal were not associated with progression at any timepoint, with nonsignificant trends in continuous data. Prior surgery was not associated with progression at 12 or 24 months, though five-year volumetric reduction was smaller in previously operated patients (-14% vs. -45.5%; p=0.037). Overall, these data align with literature showing high control across SRS and SRT, transient mid-term fluctuations attributable to pseudoprogression, and limited prognostic value of baseline imaging features, supporting vigilant volumetric follow-up while avoiding the over-interpretation of nonsignificant trends [1,5,19,25].

Clinical worsening was significantly associated with volumetric progression, with higher rates of overall decline and hearing loss at both 12 months and five years. Other symptoms showed no consistent relationship with progression. Symptom worsening showed no significant association with RT fractionation, imaging features, or prior surgery, while larger baseline tumor volume was positively associated. These findings align with prior evidence that imaging progression is the strongest predictor of clinical deterioration, particularly hearing loss, emphasizing the need for combined volumetric and audiometric follow-up [29,30]. The low rate of post-RT interventions confirms the durable tumor control achieved with SRT, regardless of regimen. The median time to salvage treatment of nearly four years further underscores the importance of sustained long-term surveillance [1,5].

The present study is strengthened by a large consecutive cohort with long follow-up, systematic volumetric and linear assessments, and consistent methodology within a specialized referral center. Limitations include its retrospective single-center design, reliance on patient-reported symptoms instead of standardized audiometry, and potential referral bias, which may restrict generalizability. As all radiologic measurements were performed by a single observer, this may introduce systematic bias but improves internal consistency for longitudinal comparisons.

## Conclusions

SRT achieved durable control of VS across SRS, HFSRT, and FSRT in this single-institution cohort, with few salvage interventions. The three-axis volumetric criterion (20% increase) outperformed the MLD 2 mm threshold and correlated strongly with contoured GTV. Early progression subsided by 24 months, and stability at 12 and 24 months predicted five-year control. Between-regimen divergence at 24 months was consistent with SRS-driven pseudoprogression, with five-year progression remaining similar across regimens. Baseline imaging offered limited prognostic value, whereas clinical worsening, mainly hearing decline, correlated with volumetric progression. Annual MRI after year 1 is generally adequate, with tighter intervals reserved for selected patients. Prospective, audiometry-standardized studies are warranted for validating hearing-related endpoints.
